# Functional and radiological outcomes of periacetabular osteotomy for hip dysplasia in patients under fifty years using a minimally invasive approach—a single surgeon series with a minimum follow up of two years

**DOI:** 10.1007/s00264-024-06094-8

**Published:** 2024-02-26

**Authors:** Karadi Hari Sunil Kumar, Kartik Bhargava, Gregory Stamp, Ajay Malviya

**Affiliations:** 1 Northumbria Healthcare NHS Foundation Trust, Ashington, UK; 2 Newcastle University, Newcastle Upon Tyne, UK

**Keywords:** Hip preservation surgery, Periacetabular osteotomy, Hip dysplasia, Outcome measures

## Abstract

**Purpose:**

We conducted a retrospective analysis of prospectively collected data to evaluate (1) the extent of surgical correction following minimally invasive periacetabular osteotomy, (2) improvements in functional outcomes and any potential predictors for favourable outcome, and (3) complications after minimally invasive periacetabular osteotomy.

**Methods:**

A total of 352 minimally invasive periacetabular osteotomy procedures were performed on 312 hip dysplasia patients between 2013 and 2020. Radiological parameters such as lateral centre edge angle, acetabular index, and Tönnis grade of arthritis were calculated. Patients also completed a range of patient reported outcome measures. Wilcoxon signed-rank tests were performed to assess for differences between patient reported outcome measures and radiological outcomes across the follow-up periods. Univariate linear regression and logistic regression were used to assess for predictors of change in functional outcome.

**Results:**

Patients had a significant correction in mean lateral centre edge angle from 17.2° to 35.3° (*p* < 0.001) and mean acetabular index from 13.2° to − 0.82°. At one year follow-up all patient reported outcome measures were significantly greater than their baseline measurements and this improvement was maintained at two years. Changes in patient reported outcome measures were independent of radiological parameters such as change in the lateral centre edge angle and acetabular index, pre-operative Tönnis grade, and patient factors such as age and sex. A total of 5.11% of patients developed post-operative complications, with four requiring posterior column fixation. Four patients (1.12%) needed a total hip replacement.

**Conclusion:**

Minimally invasive periacetabular osteotomy is a safe procedure that provides significant functional outcome improvements following surgery at six months which is maintained at two years. More than three-fourths of patients achieved improvement of iHOT-12 score beyond the minimal clinically important difference and more than half of the patients achieved substantial clinical benefit for iHOT-12 score.

## Introduction

Hip dysplasia (HD) is an abnormality of the hip joint constituted by insufficient acetabular coverage of the femoral head. If left untreated, axial overload in conjunction with reduced femoro-acetabular contact area increases the contact pressure on, and damage to, the cartilage matrix [1]. This predisposes the affected individuals to develop premature osteoarthritis of the hip, necessitating the need to undergo total hip arthroplasty at a younger age[2].

In 1998, peri-acetabular osteotomy (PAO) was established as an effective surgical technique to treat symptomatic HD while preserving hip joint anatomy [3, 4]. PAO involves a three-dimensional re-orientation of the acetabulum to provide greater acetabular coverage of the femoral head improving the load distribution, and thus delay joint degeneration and onset of secondary osteoarthritis [3]. PAO is a reproducible technique with a good functional outcome and low complication rate [5]. Several authors have reported good long-term results with a survivorship ranging from 80% at 14 years to 60% at 20 years [6–8].

More recently, minimally invasive surgical (MIS) technique has been used to modify the original Smith-Petersen approach to reduce blood loss through a reduction in soft tissue dissection and protect the lateral cutaneous nerve of the thigh. The MIS technique not only helped improve rehabilitation and facilitate early discharge but also minimised complications [9–11]. While prior longitudinal research illuminates that MIS PAO provides symptomatic benefit, there is a lack of literature on the proportion of patients achieving scores beyond the minimal clinically important difference (MCID) and achieving substantial clinical benefit (SCB), and also the radiological and demographic factors that might influence the outcome.

The objective of this study was to conduct a single-centre, single-surgeon, analysis of prospectively collected patient reported outcomes measure (PROM) of patients with HD who underwent MIS PAO and any complications associated with this procedure. We hypothesised that MIS PAO using the University College London Hospital (UCLH) technique provided functional improvement to patients with HD which could be reproduced in other centres. In particular, we wanted to report on the proportion of patients achieving PROM scores beyond MCID and achieving SCB.

## Methods

**Fig. 1:**
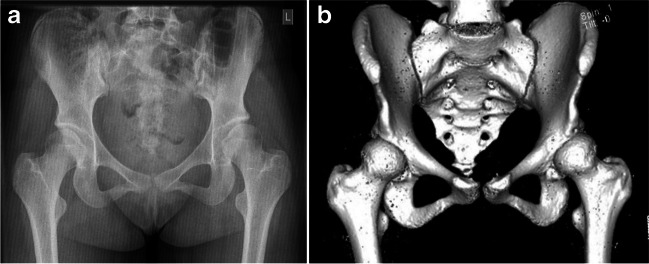
Pre-operative imaging: **a** plain AP view radiograph of a patient with acetabular dysplasia and **b** CT scan with 3D reconstruction demonstrating the extent of femoral head undercoverage

**Fig. 2:**
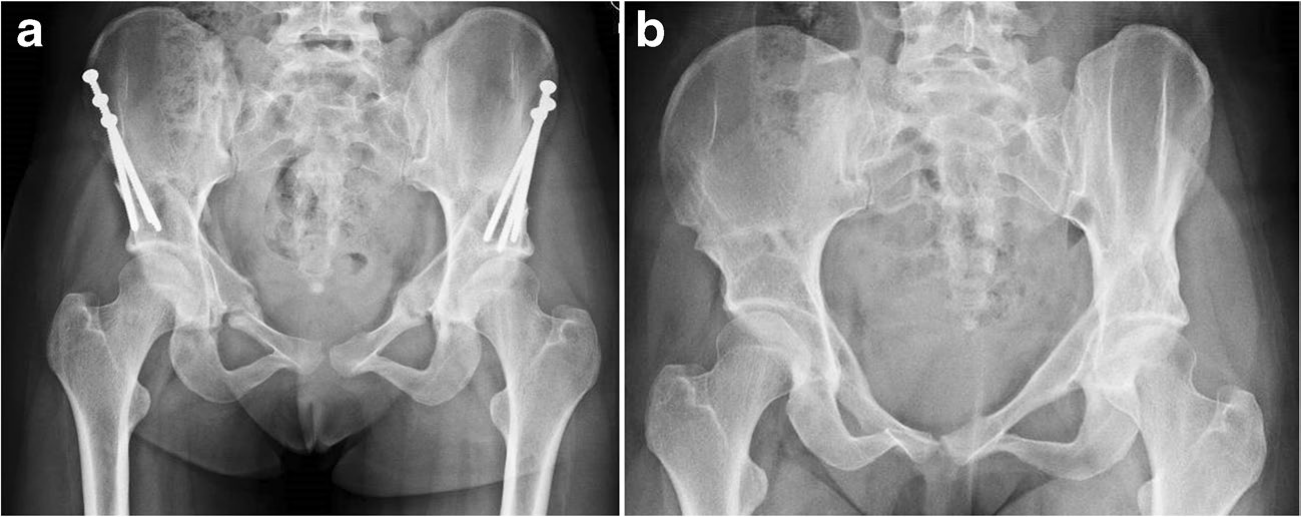
Post-operative imaging: a plain AP view radiograph after periacetabular osteotomy: **a** with screws in situ and **b** fully healed osteotomy following screw removal

We evaluated all consecutive MIS PAOs performed by the senior author (AM) between January, 2013, and March, 2020. A total of 352 MIS PAOs were performed in this period for symptomatic HD that had failed non-surgical treatment. Diagnosis was confirmed on the basis of clinical examination and imaging findings. Hip dysplasia was defined as a lateral centre edge angle (LCEA) < 25° and an acetabular index (AI) > 10° on an antero-posterior (AP) plain radiograph of the pelvis [12]. A CT scan with 3D reconstruction was performed in all cases to further evaluate the morphological abnormality if unable to make a clear diagnosis of HD on the plain radiograph [13]. A total of 352 surgeries performed on 312 patients (228 = unilateral, 62 = bilateral) were included in the final analysis. Patients who underwent bilateral surgeries at different time points were considered as separate participants for each surgery they underwent. All MIS PAOs were performed by the senior author using the UCLH technique that has been previously reported [3]. Figures 1 and 2 show the pre- and post-operative imaging of a patient who underwent a PAO in our unit. Demographic and surgical details for patients are summarised in Table 1.

**Table 1:** Demographic statistics for patients undergoing MIS-PAO

Demographic variable	*N* (%)
Sex	
Male	22 (6.2%)
Female	330 (93.8%)
Age	32.7 (sd 10.3)
BMI (body mass index)	26.8 (sd 5.2)
Follow-up	2.3–9.4 years (mean 5.2)
Hip joint	
Left sided	145
Right sided	207

The UCLH PAO technique reported by Khan et al. uses the Smith-Peterson approach with a bikini line incision just below the anterior superior iliac spine (ASIS). This is an internervous plane between sartorius and tensor facia lata (TFL) superficially and rectus femoris and gluteus medius in the deep plane. Upon identification of the ASIS, the origin of the inguinal ligament and sartorius is dissected carefully. The lateral femoral cutaneous nerve is identified and further dissection is performed lateral to it. The fascia over the TFL is incised laterally and careful dissection is performed in distal to proximal direction to proceed to the deep plane. Once in the deep plane, the interval between the iliopsoas and the rectus femoris is developed with careful elevation of the fibres of the iliocapsularis muscle from the anterior capsule. Care is taken to keep the capsule intact during the procedure. A narrow ‘Cobb’ elevator is inserted in the space and directed infero-medially anterior to the hip joint capsule to reach the ischium. The ‘Cobb’ elevator is replaced by a special angled osteotome which is used to make the ischial cut under image intensifier control. Only 50% of the ischium is osteotomised. Next the soft tissue over the superior pubic ramus is elevated and retracted with angled bi-pronged retractor and special radiolucent retractors are placed on either side of the superior pubic ramus. Osteotomy is carried under direct vision—initially the medial cut followed by a lateral cut 3–4 mm apart but taking care to be medial to the hip joint at all times. Next the iliacus is carefully elevated from the ilium subperiosteally and care taken while going over the pelvic brim to avoid damage to the pelvic vascular anastomosis. This is a step which may lead to sudden haemorrhage if not careful. The posterior column is identified and a radiolucent retractor placed to reflect the soft tissue. Posterior column osteotomy is performed under image intensifier guidance with special straight and curved osteotomes to reach the previously performed ischial osteotomy. The final cut is a transverse osteotomy of the ilium to meet the posterior column cut. A Schanz pin is inserted into the anterior inferior iliac spine to allow control of the acetabular fragment during re-orientation of the acetabulum. An AP pelvic image is obtained with image intensifier to correspond to the pre-operative radiograph so that the final correction can be achieved as planned. The acetabular fragment is fixed to the pelvis with four fully threaded screws under image intensifier guidance to ensure the screw is not penetrating the hip joint.

LCEA and AI were calculated both pre-operatively and post-operatively from the AP pelvic radiographs. Tönnis grade of arthritis was calculated for each patient [14]. If there was any doubt of the correct Tönnis grade on plain radiograph, the CT scan was assessed where available, to check the extent of joint space involvement. Patients were followed up post-operatively at six weeks, three months, six months, and one year when both clinical and radiological assessments were carried out. Patients were toe-touch weight bearing with walking aids for the first six weeks and following a review in out-patient clinic were allowed protected weight bearing for a further period of six weeks if plain radiographs were satisfactory. At three months they were allowed to wean off walking aids if there was evidence of satisfactory callus formation at the osteotomy site. Protected weight bearing was advised until three months post-operatively to prevent possible stress fracture of the posterior column. All patients were asked to complete PROM questionnaires pre-operatively and post-operatively at six month, one year, and two year follow-up. The minimum follow-up in our series was two years and the maximum was 9.4 years. The PROMs data was electronically collected independently by the UK Non-Arthroplasty Hip Registry (NAHR) and the patients who did not complete the online forms were sent postal questionnaires by the Northumbria outcomes department. All patients were consented to be included in the Registry. The process of post-operative data collection was prospective and entirely independent of the surgical team. In addition, the PROMs completion was voluntary for the patients but every patient was given the opportunity to complete the PROMs questionnaires. PROMs collected include the EuroQol-5 Dimension (EQ-5D), EuroQol-5 Dimension-Visual Analogue Scale (EQ-5D-VAS), Non-Arthritic Hip score (NAHS), short-version of International Hip Outcome Tool (iHOT-12), and University of California Los Angeles activity score (UCLA-Activity). Over the course of the follow-up period, data was collected regarding whether patients had any complications and if it required treatment.

### Ethical approval

Data for this study was extracted from the database that is prospectively collected and maintained by the Northumbria Healthcare NHS Foundation Trust for auditing and research purposes. Therefore, no ethical approval process was commenced in accordance with the http://www.hra-decisiontools.org.uk/ethics/. Due consent process was followed for inclusion of the patients in the UK Non-Arthroplasty Hip Registry (NAHR).

### Statistical analysis

Statistical analysis was performed using Statistical Package for Social Sciences (IBM SPSS Statistics, Version 28.0. Armonk, NY: IBM Corp). To ascertain the extent of clinical recovery, patients were deemed to have achieved MCID and SCB, if their iHOT-12 improved by a score of 13 and 28, respectively, over a one year period [15]. Wilcoxon signed-rank tests were performed to assess for statistical difference between PROMs and radiological outcome measures across the follow-up periods. Univariate linear regression and logistic regression were used to assess for predictors of change in functional outcome measures over 1 year. All statistical tests were performed at 95% confidence interval and *p* values < 0.05 was deemed as statistically significant.

## Results

### Change in radiological outcome for patients receiving PAO

There was good correction in LCEA from a mean of 17.2° pre-operatively to a mean of 35.3° post-operatively (*p* < 0.001). Similarly, there was satisfactory correction of the mean AI from 13.2° to − 0.82° (*p* < 0.001). Therefore, there was a mean increase in LCEA of 18.1° and decrease in AI of 14°. The Tönnis grades of the patients were as follows: grade 0, 143 (40.6%); grade 1, 162 (46%); grade 2, 47 (13.4%); and grades 3 and 4, 0.

### Evaluation of patient reported outcome measures

At the six months follow-up, patients receiving MIS PAO had significantly increased EQ5D-5L, UCLA-Activity, NAHS, and iHOT-12 scores, compared to their baseline measurements. At one year follow-up, all functional outcome scores were significantly greater than their baseline measurement, but only NAHS and iHOT-12 scores were significantly different to their six months follow-up scores. The other PROMs showed a similar improvement but were not statistically significant. At the two year follow-up, all scores apart from EQ-5D-VAS remained significantly different to their baseline, but no further statistical difference was noted between one year and two year follow-up (Table 2). At one year follow-up, 76% of patients had achieved MCID and 55.6% achieved SCB for iHOT-12 scores. This trend was maintained at 24 months with 74.6% and 59.3% achieving MCID and SCB, respectively. Functional outcome scores at each follow-up are summarised in Table 2.

**Table 2:** Functional outcome measured at each follow-up and their comparison to baseline

	Pre-op	6 months post-op	1 years post-op	2 years post-op
	Median (IQR)	Median (IQR)	Median (IQR)	Median (IQR)
EQ5D-VAS	70 (50–85)	79.5 (60–90)	80 (70–90)	80 (65–90)
EQ5D	0.53 (0.305–0.654)	0.697 (0.56–0.786)	0.71 (0.587–0.836)	0.735 (0.62–0.837)
UCLA-Activity	3 (3–5)	5 (4–6)	5 (4–7)	6 (4–7)
NAHS	49 (38–63)	76 (54.25–85)	80 (66.25–91)	80 (64–91)
iHOT-12	26 (15–37)	56.5 (37–80)	63.5 (40.75–86)	74.5 (38.5–89)

### Relationship between radiological outcomes and PROM scores

Univariate linear regression and logistic regression showed that improvement in PROMs over six months and one year was largely independent of age, sex, LCEA change, AI change, and pre-op Tönnis grade.

### Complications of PAO

A total of 18 patients (5.11%) had complications following their surgery. Out of the 352 cases of MIS PAO, 3 (0.85%) developed deep infections requiring washout and antibiotics, 11 (3.1%) had non-union (4 out of these 11 required fixation of the posterior column), 1 (0.28%) had deep vein thrombosis, 1 (0.28%) had avulsion of anterior superior iliac spine requiring fixation, 2 (0.57%) had persistent wound discharge which settled with negative pressure dressing and 3 (0.85%) had pain related to the screws. Four (1.1%) cases went on to require a total hip replacement (THR) at mean of 3.6 years (SD = 1.6). There were no cases of femoral, sciatic, or obturator nerve injury or any case of vascular injury. 

## Discussion

Minimally invasive periacetabular osteotomy provides symptomatic improvement in patients with hip dysplasia within one year post-operatively as shown by improvement in PROM scores, which is maintained at the two year follow-up. In our series 76% of patients had achieved scores beyond MCID for iHOT-12 at one year follow-up with 56% achieving SCB. To the best of our knowledge, no other study has reported the values for MCID and SCB after PAO. This is an independent-centre validation of a technique that has been previously described by Khan et al., who did not report functional outcome [3]. We report a complication rate of 5.11% with majority of these (11/352 = 3.1%) being cases of pubic or ischial (posterior column) non-union.

The proportion of patients achieving MCID in our study is less compared to 90% of patients who achieved MCID for iHOT-12 from the NAHR cohort [16]. However, the NAHR cohort reported by Holleyman et al. used a MCID score of 9 for iHOT-12 which is lower than the MCID (=13) used in our study [16]. Moreover, the study also included patients who underwent PAO for acetabular retroversion causing FAI, a cohort which we have excluded from analysis in this paper [16]. In our series the UCLA-activity score improved from a mean score of 3.99 (median = 3) pre-operatively to a mean of 5.10 at six months (median = 5) which is an improvement of 1.11 points. The patients continued to show an improvement with a mean score of 6.97 (improvement = 2.98 points) at two years (median = 6; 3 point increase). This improvement was much better than that reported by Petrie et al. who reported an average improvement of 0.6 points at final follow-up [17]. In addition, the improvement in UCLA-activity score in our cohort of patients was much better than that reported by Clohisy et al. (0.4 points) in their series of 391 PAO procedures performed by surgeons from the ANCHOR group [18]. Furthermore, in our series the NAHS improved 37.45 points from a mean of 49.78 to a mean of 77.23 at 12 months. This improvement was comparable to the improvement of 31.3 points in NAHS reported by Ramirez-Nunez et al. [5].

The improvement in outcome scores was independent of patient’s age, sex, and the degree of radiological correction achieved at surgery. At a 20-year follow-up, Steppacher et al. reported a worse outcome for patients over the age of 30 years [8]. Similarly, Matheney et al. identified age more than 35 years as one of the factors for poor outcome at a mean follow-up of 9 years [19]. In our series, at a minimum follow-up of two years (maximum follow-up of 9.4 years), we did not find any significance between post-operative outcome and age of the patient at the time of surgery. The PROM scores at two years showed a significant improvement similar to that reported in several other studies [17, 20]. The LCEA in our series improved from a mean of 17.2° pre-operatively to a mean of 35.3° post-operatively which was statistically significant. Similar corrections were achieved in other series. Fan et al. found that a post-operative LCEA of  > 38° was predictive of a poor outcome [21]. We achieved a mean LCEA of 35° which was less than that reported by Fan et al. [21]. In our cohort, there were 41 patients whose LCEA was ≥ 40°, but we did not find any correlation with poor outcome. All our patients had satisfactory correction of the LCEA irrespective of the severity of the dysplasia contrary to Novais et al. who reported a higher risk of under corrections in patients with severe dysplasia [22].

Majority of patients (305/352 = 86.9%) who underwent PAO were either Tönnis grade 0 or 1. However 13.1% (47) patients who underwent PAO in our series were Tönnis grade 2. This was because patients were keen to preserve their own joint and made an informed decision of unfavourable outcome with the risk of progression to arthritis. In this cohort of Tönnis grade 2, we did not see an increase in the progression to arthritis with majority still reporting an improvement in PROMs. Out of these Tönnis grade 2 patients, only 6.4% progressed to a THR (3/47). The other patient who underwent a THR had a Tönnis grade 1. The progression to a THR in our series compares favourably to the 8.3% conversion rate reported in a recent meta-analysis [23]. Those who progressed to have a THR had cysts in the acetabulum (*n* = 1), chondral damage (*n* = 2), and one developed cyst in the acetabulum post-operatively. Interestingly a few of the Tönnis grade 2 cases that had a small cyst in the acetabulum maintained an improvement in post-operative PROM scores. This may have been due to a decrease in cyst volume as reported by Mechlenburg et al. [24]. Furthermore, the re-directional osteotomy perhaps moved the cyst from weight bearing portion of the acetabulum to a non-weight bearing position, thereby reducing the forces at the cyst. We did not perform a post-operative CT scan so are not able to comment on the cyst size or volume in our cohort of patients. Willey et al. have shown that increasing age and presence of arthritis pre-operatively were risk factors for poor outcome and conversion to THR [25].

In our series 4 patients out of 352 (1.1%) underwent a THR for ongoing symptoms. Peters et al. reported an 8% (*N* = 4) failure of PAO at 36 months from a single centre and that all the four hips which failed were among the first 30 patients who underwent PAO in the surgeon’s learning curve [26]. In our series three out of the four patients were in the first 33 patients who underwent PAO in our unit. This brings the failure rate to 9% for the first 33 patients in our series. This does stress the importance of the learning curve in this complex procedure. Around 30–40 cases are required to progress through the learning curve in order to obtain reproducible outcomes and decrease the failure rate of this procedure. Furthermore, Larsen et al. reported a THR conversion of 5.2% at 14-year follow-up from a large series of 1385 patients [6]. Similarly, Wells et al. reported a survival rate of 92% at 15 years following PAO in their series of 238 hips [27]. The conversion to THR in our cohort is far less than that reported in the literature.

There were 11 cases of non-union/delayed-union of the posterior column or superior pubic ramus, but majority healed spontaneously with only four requiring surgical exploration, freshening of the osteotomy, and fixation with application of bone graft. These cases were associated with early weight bearing due to patient non-compliance (before 6 weeks) and incidental finding of a stress fracture of the inferior pubic ramus on the ipsilateral side. Furthermore, complication rate in our series was 5.11% which was similar than that quoted by Novais et al. (6%) and the 7% reported in a recent systematic review from our unit [10, 28] Therefore, careful pre-operative planning including measuring the width of the posterior column and specific-width osteotome are useful to minimise surgical complications [29]. It is therefore important for one to have a structured training in this procedure to go through the learning curve to achieve reproducible outcomes. In addition, a structured mentored programme especially in the initial years is useful to minimise complications while going through the learning curve [30, 31].

## Limitations

There are limitations with this study. Foremost, it is a single-centre, single-surgeon series, and while this improves expediency and ensures consistency of surgical technique, pre- and post-operative process, and rehabilitation, it does reduce generalisability of the results and therefore validity of these results as surgical techniques vary. Secondly, this is a retrospective review of prospectively collected data until 2020 and long-term follow-up is necessarily limited in more recent cases. Thirdly, although the surgical technique remained the same, there might be minor modifications as the senior surgeon went through the learning curve, which perhaps may have a small effect on the outcome. Finally, the drop-off rate in our PROMs at 12 months was almost 50% of those who completed a pre-operative questionnaire. The completion of the PROMs questionnaire is completely voluntary and all patients were invited to complete their outcomes scores but a proportion chose not to. In our cohort there are several patients who remain clinically well and are under regular follow-up but have not completed their postoperative PROMs despite being given opportunity to do so both electronically and by postal questionnaires.

## Conclusion

In this large case series of prospectively collected data, patients had significant functional outcome improvements following periacetabular osteotomy for hip dysplasia by six months, which was maintained at two years, and beyond. 76% of patients achieved minimal clinically important difference and 56% achieved substantial clinical benefit for iHOT-12 scores at one year. The minimally invasive technique for periacetabular osteotomy is reproducible and provides reliable outcomes with low complication rates. However, periacetabular osteotomy is not a benign procedure and patients need to be informed of the possibility of not improving to their desired expectations even after this major surgical intervention. This study provides guidance and further evidence of the safety and efficacy of periacetabular osteotomy for ameliorating pain and improving outcome at least in the short to medium terms in the management of hip dysplasia in symptomatic adults.

## Data Availability

Data is available on request.
